# Finding common protein interaction patterns across organisms

**Published:** 2007-01-12

**Authors:** Mirco Gerke, Erich Bornberg-Bauer, Xiaoyi Jiang, Georg Fuellen

**Affiliations:** 1 Division of Bioinformatics, Biology Department, Schlossplatz 4, D-48149 Münster, Germany;; 2 Institut für Informatik, Fachbereich Mathematik und Informatik, Einsteinstr. 62, D- 48149 Münster, Germany;; 3 Department of Medicine, AG Bioinformatics, Domagkstr. 3, D-48149 Münster, Germany

**Keywords:** protein interaction, network analysis, orthology, molecular evolution

## Abstract

Protein interactions are an important resource to obtain an understanding of cell function. Recently, researchers have compared networks of interactions in order to understand network evolution. While current methods first infer homologs and then compare topologies, we here present a method which first searches for interesting topologies and then looks for homologs. PINA (protein interaction network analysis) takes the protein interaction networks of two organisms, scans both networks for subnetworks deemed interesting, and then tries to find orthologs among the interesting subnetworks. The application is very fast because orthology investigations are restricted to subnetworks like hubs and clusters that fulfill certain criteria regarding neighborhood and connectivity. Finally, the hubs or clusters found to be related can be visualized and analyzed according to protein annotation.

## Introduction

Protein interactions play an important role in many cellular processes such as signaling, transcription regulation and multi-enzyme complexes. Interactions can be very strong as between coiled coils of myosin (Lupas et al 1991). They can be transient, as in case of bHLH (basic Helix-Loop-Helix) proteins (Bornberg-Bauer et al 1998, Amoutzias et al 2004). They can also be very unspecific, such as the ones mediated by the SH3 domain (Reiss and Schwikowski, 2004).

Over the last years many groups have studied network structures, mostly focusing on “global” features of interaction networks. Protein networks arrange in huge connected components. These have a few highly linked nodes and many sparsely linked ones. The average path from any given node to any other is short and this relationship has been termed *small world* behavior in analogy to social networks of mutual acquaintances. Models based on physical concepts have also been used to characterize global properties. For example, many networks feature a hub-like arrangement that often coincides with *scale-free* behavior (Barabasi and Oltvai, 2004).

Several groups analyzed global properties of interaction networks with the goal to answer phylogenetic questions. The endosymbiotic hypothesis was confirmed by identification and analysis of the most ancient interactions (Qin et al 2003). Conservation of protein interactions across phyla has been observed in many cases (Matthews et al 2001; Ramani and Marcotte 2003; Bork et al 2004), for example for the transcriptional network which regulates the development of the heart. It is regulated by protein interactions which have been conserved at least since the last common ancestor of fly and man (Cripps and Olson, 2002).

In a very recent strand of research, several groups have begun to systematically compare interaction networks between organisms, and of the network of one organism with itself (Matthews et al 2001; Kelley et al 2003). In the first case, orthologous subnetworks are inferred. By analogy with sequence-only analyses such as phylogeny reconstruction, “paralogous” subnetworks can be detected in the second case. The latter result from subnetwork duplications in a single species. In particular, the PathBlast tool can detect homology between linear network substructures by aligning “pathways” of prespecified length between two networks, matching interacting proteins that are similar according to BLAST (Basic local alignment search tool, Altschul et al 1997) and allowing a limited amount of mismatches and gaps. In other words, PathBlast is based on the pairwise alignment of symbols representing interacting proteins deemed similar. More recently, PathBlast has been extended to work for more than two networks simultaneously, and for non-linear substructures (Sharan et al 2005). The latter extension is based on graph theory, and it involves a large amount of similarity searches. We were interested in a lightweight approach to finding orthologous substructures shared by two interaction networks.

Based on orthologous subnetworks, the function of some of the interacting proteins may be predicted, thus extending the well known “homology implies functional analogy” paradigm (Benner et al 2000, Fuellen et al 2005). On the other hand, Yu et al (2004) demonstrated that interactions in one organism can be predicted to occur in another if the corresponding orthologs can be found (see also Huang et al 2004; Brown and Jurisica 2005). For such predictions, orthologous subnetworks are particularly useful. Last not least, the analysis of orthologous subnetworks provides insights into evolution. For example, by comparing the networks of two species, their ancestral “core” network can be estimated.

Here we present a method for the identification of orthologous subnetworks. Their identification is computationally demanding if we base the analysis on an all-against-all comparison. Therefore, as described in the first part of the paper, we calculate the clustering coefficient for all nodes in the first network, identify the nodes with an interesting connectivity pattern such as hubs and clusters, and only search for putative ortholog matches of these nodes in the second network. The second part of the paper describes several case studies, demonstrating the use of our application.

## Material and Methods

We used the protein interaction networks of Homo sapiens (homo), Mus musculus (mouse), Drosophila melanogaster (fly) and Saccharomyces cerevisiae (yeast) taken from the BIND (Biomolecular Interaction Network) database (Alfarano et al 2005) as of January/February 2005. Given two networks, the clustering coefficient of each node *i* in a network is calculated according to the standard formula (see, eg Barabasi and Oltvai, 2004)

$$c_{i}=\frac{2n_{i}}{k_{i}(k_{i}-1)}$$

where *k_i_* is the number of neighbors of node *i* and *n_i_* represents the number of connections of the neighbors of node *i* among themselves. The determination of *n_i_* is done in a straightforward way by comparing the list of neighbors of node *i* with the lists of neighbors of its neighbors, incrementing the value of *n_i_* for each match between these lists. We note that our naïve implementation only consumes a negligible fraction (much less than 1%) compared to the homology searches that follow (see below). Therefore, we did not consider any sophisticated approach (as described in, eg, Schank and Wagner, 2004) to calculating the clustering coefficient.

**Figure 1:**
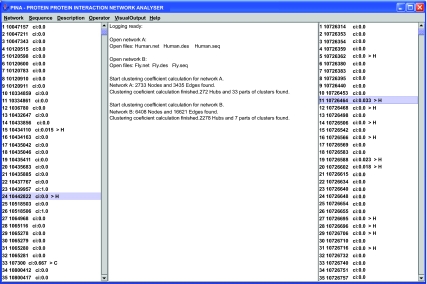
A screenshot of PINA. On the left/right, proteins of the first/second network are listed, including the clustering coefficient, and a symbol (H or C), denoting a hub or a member of a cluster, respectively. Description lines for each protein can be inspected in the logging panel in the middle, which also provides network statistics. The subnetworks corresponding to the highlighted proteins (number 24 and number 11) can be visualized and analyzed for similarity. Similarity analysis can also be done comparing the hubs and clusters of an entire network against the other network.

Given the clustering coefficient of all nodes in the networks to be compared, hubs and clusters are identified in the first network. We define a *hub* as a node in the protein interaction network, which has a low clustering coefficient (per default, its value is required to be below the *hub threshold η* = 0.1) and many neighbors (more than four by default). We define a *cluster* as a set of interacting nodes in the protein interaction network, where all nodes have a high clustering coefficient (per default, the corresponding *cluster threshold γ* is set to 0.1). Thus, to define a cluster, a breadth-first search is done until the clustering coefficient drops below *γ,* where the result of the search does not depend on its starting point. All thresholds were determined empirically. Only for the hubs and clusters of the first network, Smith-Waterman (1981) alignments are calculated to find putative orthologous proteins and, therefore, putative orthologous subnetworks in the second network. More specifically, an all-against-all comparison is performed for each hub of the first network with each hub of the second network. For the best hub-hub matches, the peripheral proteins are compared as well, in an all-against-all fashion. The two hubs are visualized together with the peripheral proteins as two subnetworks, where edges denote the interactions. All comparisons that reveal above-threshold similarity are given a link (colored red in the visualization) between nodes of the two subnetworks. In case of a cluster (in the first network), the protein with the highest clustering coefficient is compared to all proteins involved in clusters of the second network. Again, for the best matches, an all-against-all comparison is performed, comparing the proteins forming the cluster in the first network, with the proteins forming the cluster in the second network, yielding the similarity links. We do not use gapped BLAST (Altschul et al 1997), because the cost of calling BLAST externally more than outweighs the gain compared to using Smith-Waterman. The resulting similarity scores are normalized, by dividing them by the self-match similarity score of the protein used for the search. These scores are preferred to E-values or p-values, since they can be used directly as color intensities for the similarity links of our visualization (see Figure 1). For each subnetwork presented, the reciprocal search was also conducted, ie we used the putative ortholog to search the first network. If this search finds the hub or cluster that we started with, resulting in reciprocal best hits, orthology is a reasonable hypothesis (cf., eg, Remm et al 2001). If the reciprocal search finds different hits, it is possible that paralogy (duplication and subsequent speciation) is the correct hypothesis. In the current implementation such cases are not considered further.

All code is written in JAVA and provided as open source; it is available at http://www.uni-muenster.de/bioinformatics/services. PINA provides a GUI as well as command-line support. The “prefuse” toolkit (Heer et al 2005) was integrated to provide network visualization. A screenshot of our application is given in Figure 1. It should be noted that no similarity analysis can provide definite statements on orthology. To begin with, “looking back in time” is dependent on models of sequence evolution, which may be incorrect. Further, it is possible that proteins were duplicated and differentially lost after subsequent speciation events, a case known as hidden paralogy (Martin and Burg, 2002). However, hidden paralogy is unlikely to occur in parallel for many proteins, so that structures of interacting proteins are less easily mistaken as orthologs if they are not.

## Results and Discussion

We applied our orthologous subnetwork search to various networks from the BIND database, recovering observations that can be found in the literature. Three examples will be described and discussed in more detail; they were selected among all hub-hub and cluster-cluster similarity matches calculated by PINA, based on the criteria of high similarity scores and biological relevance.

**Figure 2:**
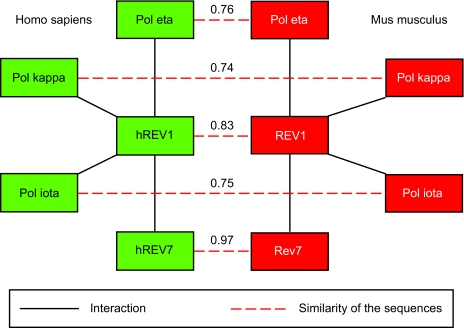
Two orthologous subnetworks found by comparing homo (left) and mouse (right). The black lines are the protein interactions and the red broken lines indicate similarity.

Figure 2 shows two subnetworks which are orthologous between Homo sapiens and Mus musculus and were found by PINA. Starting with the hub protein hREV1 (Homo), Rev1 (Mus) was found to match using Smith-Waterman. Moreover, each protein of the human subnetwork has a matching protein in the murine subnetwork; all proteins except hREV7/Rev7 are known to belong to the Y family of DNA polymerases; Rev7 belongs to the B subfamily. All these polymerases are translesion DNA synthases (TLS). Our findings confirm observations by Ohashi el al 2004, and by Guo et al 2003, both based on yeast two-hybrid assays. This is no surprise since the underlying BIND interaction data are based on these two papers. However, given the functional analysis by Guo et al and the interaction data obtained experimentally by Ohashi et al orthology, of subnetworks detected by PINA allows predicting some functional characterizations reported by Ohashi et al that were obtained before by Guo et al In both cases, Pol kappa, Pol eta and Pol iota as well as (h)Rev7 interact with the C-terminal portion of (h)Rev1, which is involved in mediating protein-protein interactions among DNA polymerases required for TLS, and the situation in human can be predicted from the one in mouse, and vice versa. Regarding the evolution of the network, we note that the Rev/Pol proteins are more similar across species than they are within a single species (see Figure 2 and Table 1), so the most parsimonious hypothesis is that the entire network already existed in the common ancestor of mouse and homo. This way, PINA has identified an evolutionary conserved subnetwork in a fully automated fashion.

Kannouche et al (2003) have shown that Pol iota and Pol eta can form (part of) a protein complex. This protein interaction is not yet included in the BIND database, and it can be predicted to exist in mouse as well. We propose that predictions of further protein interactions for already existing orthologous subnetworks are more reliable than predictions without this additional information, and we intent to use PINA for such predictions, and to evaluate these. More generally, the Rev/Pol subnetwork, like all other orthologous subnetworks found by PINA, can be used to test interactions predicted based on orthologous sequences alone.

**Figure 3:**
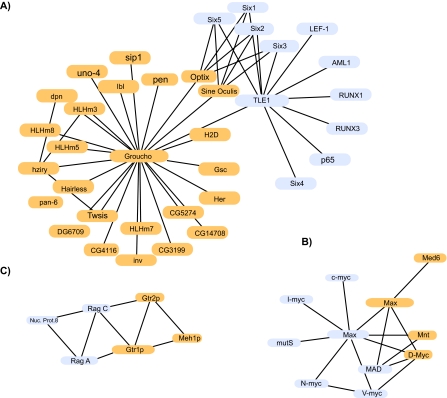
Three further orthologous subnetworks found by PINA as described in the text. Human proteins are given in light blue, their orthologs in yellow. Orthologs are from fly (panels A and B) and from yeast (panel C). The gray edges are the interactions. The red lines show high similarity between proteins from the two subnetworks, and intense red indicates highest similarity.

**Figure 4:**
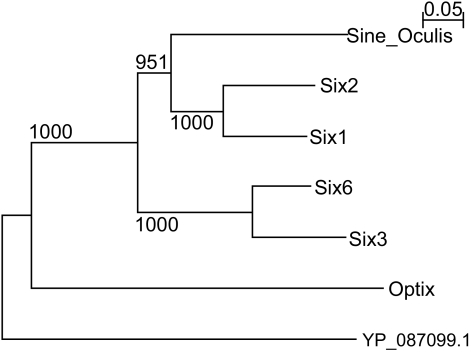
Phylogenetic tree of some proteins involved in the TLE1/Groucho network. The tree was generated by applying Neighbor-Joining to the protein sequences (Saitou and Nei, 1987), as implemented by Quicktree (Howe et al 2002). The outgroup is yeast protein YP_087099, which also features a Homeo domain. Bootstrap values are given based on 1000 replicates.

In Figure 3, three further orthologous subnetworks are described. The TLE1/Groucho networks (panel A) are not matching one-to-one as the Rev/Pol networks do. Only some proteins feature high similarity with another protein. The two hubs, Groucho and TLE1 (transducin-like enhancer protein 1), match with a high similarity score, and they are the basis of calling both subnetworks orthologous. They both play a distinct role as transcriptional repressors of a variety of other proteins involved in transcriptional regulation (Zhu et al 2002, Lopez-Rios et al 2003). Optix/Sine Oculis (SO) in fly and the human Six family are transcription factors featuring a Homeo and a Six domain; they interact with Groucho and TLE1, respectively. PINA results can be used for a functional analysis of these orthologous subnetworks, as it was done before in case of the Rev/Pol networks, and similar opportunities exist for the murine system (Zhu et al 2002). Most interestingly, however, the orthologous subnetworks also confirm hypotheses about the evolution of these networks. Similarity is strongest between Six3 and Six6, as well as between Six1 and Six2 (see Table 2), indicating recent duplications. Furthermore, Table 3 indicates that, as can already be inferred from the similarity edges in Fig. 3, Six3/6 and Optix can be assumed to have a common ancestor, just like Six 1/2 and SO. This assumption is confirmed by Gallardo et al (1999) and it is corroborated by the phylogenetic tree analysis in Fig. 4, except that our analysis places Optix next to the root of the tree. Finally, Six3/6, Optix and Six1/2/SO are probably the result of a (series of) duplications, inheriting the interaction with Groucho/TLE. In such a way, using PINA results, we can estimate the evolution of the “core” network that goes back to the ancestor of Drosophila and Homo.

Another example from the comparison of Homo and Drosophila is shown in Fig 3, panel B. In this case, the human Myc-Max-Mad transcription factor network (Luscher 2001; Partlin et al 2003; Nair and Burley, 2003; Amoutzias et al 2004) is compared to its Drosophila counterpart (Giot et al 2003). Featuring just three interactions recorded in the BIND database, the Drosophila Max protein can barely be identified as a hub. Furthermore, it is surprising that it is the human Mad protein (and not the human Max protein) that has strongest similarity links with the Drosophila Max (and Mnt) proteins. However, similarity may be misleading as far as evolutionary relationship is concerned (Koski and Golding, 2001), and a sequence-based phylogenetic tree indeed confirms the putative correct evolutionary relationship, placing the Myc and the Max proteins in one subtree each, irrespective of the species they come from (data not shown).

Our final example (Figure. 3, panel C) describes orthologous subnetworks based on matching clusters by PINA. Gtr1p and Gtr2p from yeast as well as RagA and RagC from human are nuclear G proteins (Nakashima et al 1999; Sekiguchi et al 2001). G proteins are regulatory GTP hydrolases, which function as molecular switches and are involved in cell development. Rag proteins are known to feature homology with Gtr proteins, and to interact with each other, two observations that are also found by PINA. Additionally, our orthologous subnetworks display them together with two uncharacterized proteins. As soon as any one of these is studied in detail, we expect to learn about the other as well.

Naturally, due to our restriction to “interesting” network topologies (hubs, clusters), our method cannot normally find all orthologous subnetworks. In brief, there are two extreme cases: On one hand, if the network clusters in one giant well-connected component, so that we have only one cluster and no hubs, then our approach will just do an all-against-all comparison, and find the “usual” putative orthologs. On the other hand, if the network is very disconnected, our approach yields very distinctive results, focusing on hubs as well as smaller well-connected clusters as candidates for orthologous subnetworks.

## Conclusions

The results presented here show how a scheme first looking for the topology of the protein-interaction network and then doing homology searches for the proteins involved can enhance our view on network evolution. Demonstrating the potential of comparative protein interaction network analysis (phyloproteomics), investigations of protein function and prediction of further interactions can be based on the orthologous subnetworks that we are able to identify using PINA. Future work includes comparative analyses across more than two species, and a formal approach towards reconstructing the evolution of interaction networks based on such data.

## Figures and Tables

**Table 1:** Similarity scores for the Rev/Pol subnetwork.

Homo	Pol eta	Pol kappa	hREV1	Pol iota	hREV7
Pol eta	1.00	0.10	0.08	0.13	0.01
Pol kappa	0.08	1.00	0.09	0.09	0.01
hREV1	0.05	0.06	1.00	0.06	0.01
Pol iota	0.12	0.11	0.10	1.00	0.01
hREV7	0.04	0.05	0.05	0.04	1.00

**Mouse**	**Pol eta**	**Pol kappa**	**Rev1**	**Pol iota**	**Rev7**

Pol eta	1.00	0.10	0.09	0.13	0.01
Pol kappa	0.09	1.00	0.09	0.10	0.01
Rev1	0.05	0.06	1.00	0.05	0.01
Pol iota	0.13	0.12	0.09	1.00	0.01
Rev7	0.03	0.05	0.04	0.04	1.00

**Table 2:** Similarity values of the TLE1 (top) and Groucho (bottom) subnetwork.

	Six1	Six2	Six3	Six6	TLE1
**Six1**	1.00	0.75	0.48	0.48	0.03
**Six2**	0.73	1.00	0.47	0.47	0.06
**Six3**	0.41	0.42	1.00	0.60	0.03
**Six6**	0.56	0.58	0.82	1.00	0.04
**TLE1**	0.01	0.02	0.01	0.01	1.00

		**Sine Oculis**	**Optix**		**Groucho**

**Sine Oculis**		1.00	0.30		0.03
**Optix**		0.26	1.00		0.05
**Groucho**		0.02	0.03		1.00

**Table 3:** Similarity values of members of the TLE1 subnetwork versus members of the Groucho subnetwork.

Sine Oculis	Six1	0.61
Sine Oculis	Six2	0.57
Sine Oculis	Six3	0.43
Sine Oculis	Six6	0.54
Optix	Six1	0.44
Optix	Six2	0.44
Optix	Six3	0.49
Optix	Six6	0.65
TLE1	Groucho	0.58

## References

[b1-ebo-2-45] Alfarano C, Andrade CE, Anthony K (2005). The Biomolecular Interaction Network Database and related tools 2005 update. Nucleic Acids Res.

[b2-ebo-2-45] Altschul SF, Madden TL, Schaffer AA, Zhang J, Zhang Z, Miller W, Lipman DJ (1997). Gapped BLAST and PSI-BLAST: a new generation of protein database search programs. Nucleic Acids Res.

[b3-ebo-2-45] Amoutzias GD, Robertson DL, Oliver SG, Bornberg-Bauer E (2004). Convergent evolution of gene networks by single-gene duplications in higher eukaryotes. EMBO Rep.

[b4-ebo-2-45] Barabasi AL, Oltvai ZN (2004). Network biology: understanding the cell’s functional organization. Nat Rev Genet.

[b5-ebo-2-45] Benner SA, Chamberlin SG, Liberles DA, Govindarajan S, Knecht L (2000). Functional inferences from reconstructed evolutionary biology involving rectified databases-an evolutionarily grounded approach to functional genomics. Res Microbio.

[b6-ebo-2-45] Bork P, Jensen LJ, von Mering C, Ramani AK, Lee I, Marcotte EM (2004). Protein interaction networks from yeast to human. Curr Opin Struct Biol.

[b7-ebo-2-45] Bornberg-Bauer E, Rivals E, Vingron M (1998). Computational approaches to identify leucine zippers. Nucleic Acids Res.

[b8-ebo-2-45] Brown KR, Jurisica I (2005). Online predicted human interaction database. Bioinformatics.

[b9-ebo-2-45] Cripps RM, Olson EN (2002). Control of cardiac development by an evolutionarily conserved transcriptional network. Dev Biol.

[b10-ebo-2-45] Fuellen G, Spitzer M, Cullen P, Lorkowski S (2005). Correspondence of function and phylogeny of ABC proteins based on an automated analysis of 20 model protein datasets. Proteins.

[b11-ebo-2-45] Gallardo ME, Lopez-Rios J, Fernaud-Espinosa I, Granadino B, Sanz R, Ramos C, Ayuso C, Seller MJ, Brunner HG, Bovolenta P, Rodriguez de Cordoba S (1999). Genomic cloning and characterization of the human homeobox gene SIX6 reveals a cluster of SIX genes in chromosome 14 and associates SIX6 hemizygosity with bilateral anophthalmia and pituitary anomalies. Genomics.

[b12-ebo-2-45] Giot L, Bader JS, Brouwer C (2003). A protein interaction map of Drosophila melanogaster. Science.

[b13-ebo-2-45] Guo C, Fischhaber PL, Luk-Paszyc MJ, Masuda Y, Zhou J, Kamiya K, Kisker C, Friedberg EC (2003). Mouse Rev1 protein interacts with multiple DNA polymerases involved in translesion DNA synthesis. EMBO J.

[b14-ebo-2-45] Heer J, Card SK, Landay JA (2005). prefuse: a toolkit for interactive information visualization. CHI 2005, Human Factors in Computing Systems.

[b15-ebo-2-45] Huang TW, Tien AC, Huang WS, Lee YC, Peng CL, Tseng HH, Kao CY, Huang CY (2004). POINT: a database for the prediction of protein-protein interactions based on the orthologous interactome. Bioinformatics.

[b16-ebo-2-45] Kannouche P, Fernandez de Henestrosa AR, Coull B, Vidal AE, Gray C, Zicha D, Woodgate R, Lehmann AR (2003). Localization of DNA polymerases eta and iota to the replication machinery is tightly co-ordinated in human cells. EMBO J.

[b17-ebo-2-45] Kelley BP, Yuan B, Lewitter F, Sharan R, Stockwell BR, Ideker T (2004). PathBLAST: a tool for alignment of protein interaction networks. Nucleic Acids Res.

[b18-ebo-2-45] Koski LB, Golding GB (2001). The closest BLAST hit is often not the nearest neighbor. J Mol Evol.

[b19-ebo-2-45] Lopez-Rios J, Tessmar K, Loosli F, Wittbrodt J, Bovolenta P (2003). Six3 and Six6 activity is modulated by members of the groucho family. Development.

[b20-ebo-2-45] Lupas A, Van Dyke M, Stock J (1991). Predicting coiled coils from protein sequences. Science.

[b21-ebo-2-45] Luscher B (2001). Function and regulation of the transcription factors of the Myc/Max/Mad network. Gene.

[b22-ebo-2-45] Martin AP, Burg TM (2002). Perils of paralogy: using HSP70 genes for inferring organismal phylogenies. Syst Biol.

[b23-ebo-2-45] Matthews LR, Vaglio P, Reboul J, Ge H, Davis BP, Garrels J, Vincent S, Vidal M (2001). Identification of potential interaction networks using sequence-based searches for conserved protein-protein interactions or “interologs”. Genome Res.

[b24-ebo-2-45] Nair SK, Burley SK (2003). X-ray structures of Myc-Max and Mad-Max recognizing DNA. Molecular bases of regulation by proto-oncogenic transcription factors. Cell.

[b25-ebo-2-45] Nakashima N, Noguchi E, Nishimoto T (1999). Saccharomyces cerevisiae putative G protein, Gtr1p, which forms complexes with itself and a novel protein designated as Gtr2p, negatively regulates the Ran/Gsp 1p G protein cycle through Gtr2p. Genetics.

[b26-ebo-2-45] Ohashi E, Murakumo Y, Kanjo N, Akagi J, Masutani C, Hanaoka F, Ohmori H (2004). Interaction of hREV1 with three human Y-family DNA polymerases. Genes Cells.

[b27-ebo-2-45] Partlin MM, Homer E, Robinson H, McCormick CJ, Crouch DH, Durant ST, Matheson EC, Hall AG, Gillespie DA, Brown R (2003). Interactions of the DNA mismatch repair proteins MLH1 and MSH2 with c-MYC and MAX. Oncogene.

[b28-ebo-2-45] Qin H, Lu HH, Wu WB, Li WH (2003). Evolution of the yeast protein interaction network. Proc Natl Acad Sci U S A.

[b29-ebo-2-45] Ramani AK, Marcotte EM (2003). Exploiting the co-evolution of interacting proteins to discover interaction specificity. J Mol Biol.

[b30-ebo-2-45] Reiss DJ, Schwikowski B (2004). Predicting protein-peptide interactions via a network-based motif sampler. Bioinformatics.

[b31-ebo-2-45] Remm M, Storm CE, Sonnhammer EL (2001). Automatic clustering of orthologs and in-paralogs from pairwise species comparisons. J Mol Biol.

[b32-ebo-2-45] Schank T, Wagner D (2004). Approximating Clustering-Coefficient and Transitivity. Technical Report.

[b33-ebo-2-45] Sekiguchi T, Hirose E, Nakashima N, Ii M, Nishimoto T (2001). Novel G proteins, Rag C and Rag D, interact with GTP-binding proteins, Rag A and Rag B. J Biol Chem.

[b34-ebo-2-45] Sharan R, Suthram S, Kelley RM, Kuhn T, McCuine S, Uetz P, Sittler T, Karp RM, Ideker T (2005). Conserved patterns of protein interaction in multiple species. Proc Natl Acad Sci U S A.

[b35-ebo-2-45] Smith TF, Waterman MS (1981). Identifi cation of common molecular subsequences. J Mol Biol.

[b36-ebo-2-45] Yu H, Luscombe NM, Lu HX, Zhu X, Xia Y, Han JD, Bertin N, Chung S, Vidal M, Gerstein M (2004). Annotation transfer between genomes: protein-protein interologs and protein-DNA regulogs. Genome Res.

[b37-ebo-2-45] Zhu CC, Dyer MA, Uchikawa M, Kondoh H, Lagutin OV, Oliver G (2002). Six3-mediated auto repression and eye development requires its interaction with members of the Groucho-related family of co-repressors. Development.

